# The micromorphology of *Trichoderma reesei* analyzed in cultivations on lactose and solid lignocellulosic substrate, and its relationship with cellulase production

**DOI:** 10.1186/s13068-016-0584-0

**Published:** 2016-08-09

**Authors:** Vera Novy, Maximilian Schmid, Manuel Eibinger, Zdenek Petrasek, Bernd Nidetzky

**Affiliations:** 1 Institute of Biotechnology and Biochemical Engineering, Graz University of Technology, NAWI Graz, Petersgasse 12/I, 8010 Graz, Austria; 2 Austrian Centre of Industrial Biotechnology, Petersgasse 14, 8010 Graz, Austria

**Keywords:** *Trichoderma reesei*, Morphology, Cellulase production, Lignocellulose, Wheat straw, Lactose

## Abstract

**Background:**

*Trichoderma reesei* is the principal producer of cellulolytic enzymes. Because of the strong influence on the enzyme production, the morphology of the filamentous fungi is a key parameter for process optimization. For cost-effective production of cellulolytic enzymes, the cultivation of *T. reesei* is performed on lignocellulosic waste streams. These insoluble substrates prevent the application of the conventional light microscopy for the analysis of fungal morphology. Here, we present a novel method for the micromorphological analysis based on confocal laser-scanning microscopy (CLSM) and the computer-aided image analysis. This method enabled the quantification of the dimensions of the single cell (intercalary length and cell width) and the degree of branching in cultivations on the industrially relevant substrates wheat straw and lactose. The micromorphology of two *T. reesei* strains, QM9414 and a carbon catabolite derepressed *cre1* knockout mutant (Δcre1), was analyzed in dependence of substrate, inoculation method, and agitation velocity.

**Results:**

*Trichoderma reesei* strain Δcre1 formed shorter cells (10.09 µm) on average and developed more ramified mycelia (0.36 branches/cell) than strain QM9414 (12.03 µm, 0.22 branches/cell). Cultivated on wheat straw, the average cell length of QM9414 (10.87 µm) and Δcre1 (9.74 µm) was 10 and 21 % shorter as compared to reference cultivations on lactose. When inoculation was done with spores as compared to hyphal biomass, cell lengths of QM9414 (10.97 µm) and Δcre1 (9.10 µm) were on average about 20 % shorter. Strain performance was evaluated in protein concentration and total cellulase activity, which varied between 0.69 and 2.31 FPU/mL for Δcre1 and between 0.84 and 1.64 FPU/mL for QM9414. The cell length exhibited slightly negative correlation with the protein (regression coefficient −0.04 g/(L µm), *R* ^2^ 0.33) and the cellulase (−0.30 FPU/(mL µm), *R* ^2^ 0.53) production.

**Conclusions:**

The dimensions of the single cell of *T. reesei* were dependent on strain background, substrate used and process conditions applied. Micromorphological changes were correlated semi-quantitatively with the efficiency of enzyme production. In providing a process analytical tool for enzyme production by *T. reesei* on lignocellulosic substrate, this study has relevance for the characterization and optimization of a critical step in the overall saccharification process.

**Electronic supplementary material:**

The online version of this article (doi:10.1186/s13068-016-0584-0) contains supplementary material, which is available to authorized users.

## Background

The commercial viability of biorefinery processes is heavily dependent on the costs of cellulolytic enzymes [1]. To successfully utilize lignocellulose as carbon source, it is, therefore, necessary to intensify the enzyme production process [1, 2]. Based on the fast biomass growth and the ability to secrete high amounts of proteins, the filamentous fungus *Trichoderma reesei* has become the principal producer of cellulolytic enzymes [3]. However, the complex morphology of the fungus presents a major challenge in process development [4–6]. *Trichoderma reesei,* such as other filamentous fungi, e.g., *Aspergillus* spp., can develop into various micro- and macromorphologic states [4–6]. The micromorphology describes the dimensions of the cells and the hyphae, as well as the degree of branching and the total number of tips [4, 6]. The macromorphology of the fungi can be broadly classified into pellets and freely dispersed mycelia [5, 6]. By employing wide-field light microscopy, it has been shown that the micro- and the macromorphology of cellulase-producing *T. reesei* is dependent on the carbon source [7–9], the composition of the culture medium [7, 8], the pH [10], the size of the inoculum [8], and the intensity of agitation [7, 11]. With the aim of quantifying morphological changes, the acquired images were analyzed towards the projected area of free and entangled mycelia, as well as the dimensions of the hyphae and the level of branching [7, 11–13]. This enabled the correlation of the micromorphology with the cellulase productivity [7, 11, 12], and showed that the micromorphology was a key factor for the process analysis and optimization. However, there are clear limitations with this approach. First, the efficient and cost-effective production of enzymes often requires the cultivations of *T. reesei* on hemicellulosic and cellulosic waste streams with high solid loadings [14]. These substrates are insoluble and often bulky or fibrous, and preclude the application of wide-field light microscopy. Consequently, studies have been exclusively conducted on model substrates, e.g., Avicel or Solka Floc cellulose [7–9, 12, 13, 15, 16]. Second, the fungal hyphae form filamentous 3D networks. Wide-field light microscopy, however, only provides images representing a 2D projection of this network. Only a thin section of the sample, corresponding to the depth of focus, is imaged sharply; the regions above and below this section are unsharp. This complicates the quantitative image analysis. Furthermore, as all structures are projections into the image plane, no axial distances can be measured.

In this study, we present a new quantitative method for the analysis of fungal micromorphology. Using confocal laser-scanning microscopy (CLSM) and an in-house developed program for image analysis, it was possible to analyze and quantify the dimensions of the single cells and the degree of branching in cultivations on wheat straw and lactose. Based on its abundance and low price, wheat straw constitutes a promising renewable carbon source in Europe [10] which can be used for cellulase production by *T. reesei*. Lactose also induces the expression of the complete set of hemicellulases and cellulases in *T. reesei* [17], although the level of expression is much lower as compared to wheat straw [18]. Lactose is the preferred carbon source when a soluble substrate is required [19]. The micromorphology was analyzed in two different *T. reesei* strains; the reference strain QM9414 [20] and strain Δcre1. The latter has the *cre1* transcription factor removed from the QM9414 genome and is, therefore, incapable of carbon catabolite repression. Based on the higher cellulase productivity, *cre1* knockout mutants are the preferred strains for efficient enzyme production [20–23]. Removal of the *cre1* gene in *T. reesei* [22] or of the orthologue *creA* gene in *A. nidulans* [24] has also been shown to result in a significant distortion of the colony morphology. However, micromorphological changes of the *cre1* or *creA* knockout mutants have not been analyzed, and studies have been solely conducted on model substrates [22, 24]. Therefore, this prompted us to perform a comparative analysis of the micromorphology of *T. reesei* QM9414 parent and Δcre1 knockout strains in cultivations on a “real” lignocellulosic substrate.

By employing the novel CLSM-based method, the dimensions of the single cells were analyzed in dependence of strain, carbon source, inoculation method, and agitation velocity. The micromorphological changes were then correlated with the protein secretion and the enzyme production.

## Methods

### Chemicals and media

Unless stated otherwise, all chemicals were from Carl Roth  +  Co KG (Karlsruhe, Germany). Potato-dextrose-agar (PDA) plates were prepared as described by the manufacturer. Mineral media (MM) contained 5 g/L yeast extract, 5 g/L KH_2_PO_4_, 3.75 g/L (NH_4_)_2_SO_4_, 0.3 g/L MgSO_4_  ×  7H_2_O, 0.3 g/L CaCl_2_  ×  2H_2_O, 100 µL/L Antifoam 204 (Sigma-Aldrich, St. Louis, MO, USA), 0.5 g/L rapeseed oil and 1 mL/L trace elements (5 g/L FeSO_4_  ×  7H_2_O, 1.6 g/L MnSO_4_  ×  H_2_O, 1.4 g/L ZnSO_4_  ×  7H_2_O, 2 g/L CoCl_2_  ×  6H_2_O, 15 g/L EDTA disodium chloride salt). Tween80 supplemented cultivations additionally contained 0.1 % (w/w) Tween80. As carbon source 10 g/L glucose, 14 g/L lactose or 14 g dry mass/L wheat straw was used as indicated. The wheat straw was pretreated by steam explosion. A detailed description of the pretreatment method and the composition of the feedstock can be found elsewhere [2].

### Strains and maintenance

The *T. reesei* mutant strains QM9414 and Δcre1 were utilized. Both strains were kindly provided by Prof. Bernhardt Seiboth (Vienna University of Technology, Vienna, Austria) and stored as spores in 15 % glycerol at −80 °C.

### Shaken flask cultivations

All cultivations were conducted in 300 mL wide mouth Erlenmeyer flasks closed with cotton wool stoppers. Incubation was at 28 °C and 190 or 150 rpm in a Certomat BS-1 orbital incubator shaker (Sartorius AG, Göttingen, Germany). Two different inoculation methods were applied in this study, either with a piece of overgrown agar or with spores. For the former, 100 µL of glycerol stock was streaked on PDA plates and incubated at 30 °C for 3 days. Subsequently, a piece (~2 cm^2^) of overgrown agar was cut out and transferred to the cultivation medium. For the inoculation with spores, PDA plates were incubated until sporulation occurred (12–24 days). Spores were then harvested by washing the plates with a Triton X-100 solution [0.1 % (w/v), Sigma]. The spore concentration was determined with a Neubauer counting chamber. The spore solution was added to the cultivation medium to the final concentration of 10^5^ spores/mL. Cultivations were either inoculated directly or from a preculture. Precultures were performed in glucose-based MM. Inoculation was with the two methods described above. Incubation of the precultures was at 28 °C and 190 rpm (Certomat) for 48 h. The biomass was harvested by centrifugation (15,700*g*, 4 °C for 10 min, Eppendorf 5415 R, Eppendorf, Hamburg, Germany) and 40 mg of it were used to inoculate the main cultivation.

### Sampling, determination of protein concentration and cellulase activity

Samples were taken for the analysis of micromorphology and enzyme production. For image analysis, the fungal mycelia was fixated with 10 % (v/v) formaldehyde solution (100 g/L) and stored at 4 °C. For the analysis of total cellulase activity (FPU/mL) and protein concentration, samples were centrifuged (15,700*g*, 4 °C for 10 min, Eppendorf 5415 R, Eppendorf, Hamburg, Germany) and the supernatant stored at 4 °C. For the determination of protein concentration, proteins were precipitated and quantified against a BSA standard utilizing the Roti-Quant kit (Roth) and following the manufacturer’s instructions. FPU activity was measured following the International Union of Pure and Applied Chemistry (IUPAC) recommendations [25]. β-Glucosidase activity was determined using para-nitrophenyl-β-d-glucopyranoside as substrate as described elsewhere [2].

### CLSM image acquisition

For the CLSM analysis, the samples were prepared as follows. The fixated biomass was washed with 50 mM sodium carbonate–bicarbonate buffer (pH 9.2) and then transferred to an object slide. The samples were immersed with a Calcoflour White solution (0.01 g/L, Sigma) and incubated for 5 min. The samples were imaged on a Leica TCS SPE confocal laser-scanning microscope (Leica Microsystems, Wetzlar Germany) using a 40×/1.15 oil objective. The excitation wavelength was 405 nm, and the emission was detected in the spectral range 420–530 nm, matching the spectral characteristics of the dye Calcofluor White. Typically, a series of 60–100 confocal images at different axial positions (*z*-stack) was recorded, yielding a 3D representation of the sample. The spacing between neighboring images in the axial direction (*z*) was 0.46 µm, resulting in the total imaged volume depth of 28–46 µm. The lateral size of the images was 183 × 183 µm (1024 × 1024 pixels).

An interactive program with a graphical user interface was written in MATLAB (MathWorks, Natick, MA, USA) to analyze the morphology of the fungi from the 3D confocal images. The software displays the images and allows scrolling through the third (axial) dimension. The user is required to identify the center positions of the septa between the cells by a mouse click, to connect the pairs of septa defining a cell, and to mark branching cells. In this way, a 3D skeleton of the fungal hyphae is defined. The software then automatically calculates the cell lengths and, on basis of the fluorescence intensity of cell walls, the cell widths. The results are saved in a text file for further analysis. An example of the interface is given in Additional file 1. Examples of the cell length distribution are depicted in Additional file 2.

## Results and discussion

### Macromorphology of strains QM9414 and Δcre1

When *T. reesei* strains QM9414 and Δcre1 were cultivated on lactose, a distinct difference in the macromorphology was observed (Additional file 3). Whereas Δcre1 developed into small and dense pellets, QM9414 formed larger and loose pellets (Additional file 3). On wheat straw, both strains showed dispersed growth. The straw fibers likely prevented the aggregation of hyphae and spores, which is the initial step in pellet formation [26]. A similar effect has been described for the addition of micro-particles to cultivations by filamentous fungi [27, 28]. As expected, the high solid content of the cultivations precluded analysis of fungal morphology in wheat straw cultivations by light microscopy (Additional file 3).

### CLSM image acquisition and analysis for the quantification of fungal micromorphology

CLSM features many advantages over wide-field microscopy, which are essential for the analysis and accurate quantification of the fungal micromorphology in cultivations on insoluble and soluble substrates. First, based on the specific pattern of the staining and the resulting fluorescence signal, structural differences between the fungal biomass and the plant material can be discerned. This made it possible to differentiate the hyphae from the wheat straw fibers, which would not be possible in the conventional light microscope images (Additional file 4). Second, CLSM yields an image of a thin sample section coinciding with the objective focal plane, without unfocused contributions from other regions of the sample. The absence of out-of-focus light results in a higher contrast and sharper images, facilitating the quantitative analysis. Third, by recording images at a range of axial (*z*) positions, a set of *xy* images is obtained which contains full 3D information (*xyz*). This set of images (“*z*-stack”) allows length measurements of cells oriented not only horizontally, but also at any angle with respect to the focal plane. In contrast to wide-field light microcopy studies, where only the projected hyphal dimensions could be measured [7, 11–13, 15, 29], CLSM, therefore, enables the quantification of the *actual* dimensions of the cells. A MATLAB program has been developed for the quantification of the geometry of the fungal network from the CLSM images. The program interactively displays the images of different *z* sections and allows the user to identify the fungal cells in 3D. Based on the septa positions identified by the user and on the fluorescence intensity of the cell walls, the program automatically calculates the intercalary length and the cell width, respectively. This semiautomated approach facilitated highly accurate length and width measurements. An example of the interface and the 3D hyphae skeleton is given in Additional file 1.

### Analysis and quantification of fungal micromorphology in dependence of strain, carbon source, and cultivation conditions and its correlation to enzyme production

The two strains, QM9414 and Δcre1, were cultivated on wheat straw and lactose. Cultivations were started by adding a piece of overgrown agar or through inoculation with spores to a count of approximately 10^5^/mL. The protein concentrations were measured over time, and the resulting time courses are depicted in Additional file 5. Regardless of the cultivation conditions, both the strains showed a lag phase of approximately 50 h. Afterwards, the protein concentrations started to increase until it plateaued at approximately ~200 h of cultivation. After ~250 h, the protein concentrations increased again. The final increase was probably caused by cell proteins being liberated by autophagy [30]. Samples for the analysis of the micromorphology and enzyme production were, therefore, taken after 8 days of incubation when the protein concentration was highest and before induction of autophagy (Additional file 5; [30]).

Biomass growth of strains QM9414 and Δcre1 was also analyzed (Additional file 5). After 8 days of cultivations, the wet cell weight produced by Δcre1 was approximately 1.3-fold higher than that produced by QM9414. Furthermore, both strains produced 1.7-fold more biomass when cultivations were inoculated with a piece of overgrown agar than with spores (Additional file 5). To equalize starting conditions, precultures were therefore performed in glucose media. Results presented below are from the main cultures, which were inoculated from these precultures.

Table 1 summarizes the cell lengths and widths, the numbers of branches formed per cell, the protein concentrations, and the volumetric cellulase activities from the presented cultivation conditions.

**Table 1:** Fungal micromorphology, protein concentration and cellulase activity obtained in cultivations on lactose and wheat straw by *T. reesei* strains Δcre1 and QM9414

	Cell length (µm)	Cell width (µm)	Branches/cell (-)	FPU/mL	Protein (g/L)
*T. reesei* Δcre1					
Wheat straw					
Agar-190	10.21 ± 3.43	3.29 ± 1.34	0.40	1.74	0.59
Agar-150	10.08 ± 3.17	2.80 ± 0.80	0.41	0.69	0.33
Spore-190	8.82 ± 4.18	3.85 ± 0.89	0.38	1.88	0.57
Spore-150	9.75 ± 2.92	3.47 ± 1.06	0.48	1.82	0.30
Agar-190-Tw	11.73 ± 3.81	3.60 ± 1.51	0.40	1.27	0.38
Spore-190-Tw	7.87 ± 2.21	3.98 ± 1.09	0.08	2.31	0.59
*Average value*	9.74 ± 1.32	3.50 ± 0.42	0.36 ± 0.14	n.c.	n.c.
Lactose					
Agar-190	11.75 ± 4.12	3.01 ± 0.77	0.43	n.d.	0.27
Agar-150	11.67 ± 4.64	3.20 ± 0.56	0.25	n.d.	0.26
Spore-190	10.51 ± 3.20	3.42 ± 0.97	0.28	n.d.	0.30
Spore-150	8.55 ± 3.32	2.91 ± 1.01	0.50	n.d.	0.26
*Average value*	10.62 ± 1.49	3.13 ± 0.23	0.36 ± 0.12	n.c.	n.c.
*T. reesei* QM9414					
Wheat straw					
Agar-190	11.84 ± 3.43	3.05 ± 0.94	0.05	0.88	0.64
Agar-150	10.21 ± 2.92	3.04 ± 1.03	0.40	0.84	0.31
Spore-190	10.42 ± 3.15	3.32 ± 1.23	0.01	1.45	0.58
Spore-150	10.20 ± 3.21	2.35 ± 0.95	0.26	1.64	0.26
Agar-190-Tw	12.47 ± 3.83	2.67 ± 1.27	0.06	0.77	0.43
Spore-190-Tw	10.09 ± 2.67	3.30 ± 1.41	0.40	1.14	0.44
*Average value*	10.87 ± 1.02	2.95 ± 0.38	0.16 ± 0.15	n.c.	n.c.
Lactose					
Agar-190	16.74 ± 5.40	2.59 ± 0.69	0.25	n.d.	0.09
Agar-150	14.24 ± 4.11	3.03 ± 1.20	0.31	n.d.	0.25
Spore-190	13.53 ± 4.82	2.82 ± 0.64	0.23	n.d.	0.09
Spore-150	10.59 ± 3.47	3.32 ± 0.53	0.49	n.d.	0.26
*Average value*	13.77 ± 2.19	2.94 ± 0.31	0.32 ± 0.09	n.c.	n.c.

Volumetric cellulase activity (FPU/mL) and protein concentration represent mean values of two experiments. Dimensions of the cells represent mean values and the spread from 40 cells analyzed from one representative CLSM image. The branches per cell were analyzed in the same image and represent the value of 60–100 cells

*n.c.* not calculated, *n.d.* not detectable

#### *Trichoderma reesei* strains QM9414 and Δcre1

When cultivated on wheat straw, *T. reesei* strains QM9414 and Δcre1 produced between 0.69 and 2.31 FPU/mL in 8 days (Table 1). Both the strains showed significant variations in the secreted total cellulase activity. Under the same cultivation conditions, Δcre1 always produced a higher volumetric cellulase activity than QM9414 (Table 1). Because QM9414, in contrast to Δcre1, is carbon catabolite repressed, this result was as expected [20–23]. On lactose, both the strains only produced basal amounts of cellulases, and the resulting volumetric activity did not reach the lower detection limit of the FPU assay. Therefore, the total protein concentration was measured in the cultivation supernatant. Within the different experimental setups, no significant variations between strain QM9414 and Δcre1 were observed (Table 1).

It has been shown in studies analyzing the proteome and the secretome of cellulase-producing *T. reesei* strains that the relative composition of the produced hemicellulolytic and cellulolytic enzyme mixture was changing in dependence of carbon source, cultivation conditions, and genetic strain background (cf [18, 31, 32]). The increase in total cellulase activity observed for Δcre1 as compared to QM9414 in cultivations on wheat straw might, therefore, have been caused by the change of the ratios of the enzyme activities rather than by an increase in protein secretion [32]. Consistently, we found that the ratio of total cellulase activity (FPU/mL) to β-glucosidase activity (CBU/mL) was changing in dependence of strain and experimental setup (Additional file 6). However, a more detailed analysis of the protein and enzyme mixture secreted by the *T. reesei* strains was beyond the scope of this research. Therefore, the total cellulase activity was used as benchmark for comparison throughout the current study. Furthermore, we show, hereinafter, that the changes in micromorphology can be related to the protein concentration independent of the strain used. Lack of knowledge of the enzyme composition was not considered to restrict the relevance of the evidence presented.

**Fig. 1:**
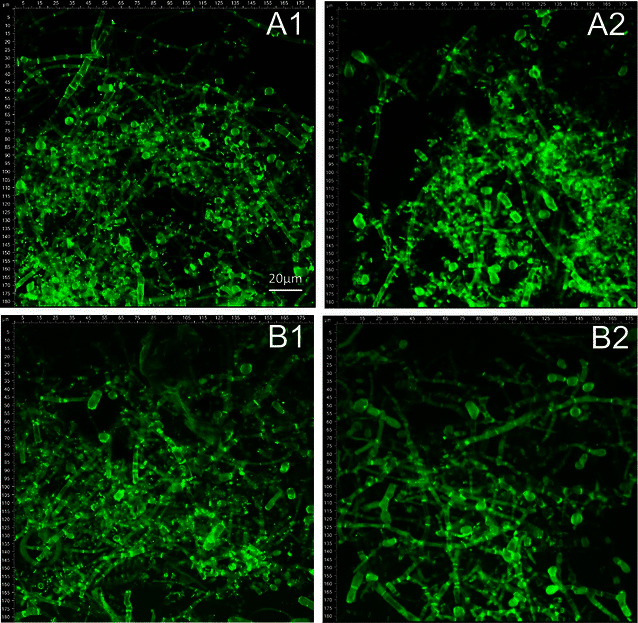
CLSM images of wheat straw cultivations by *T. reesei* strains QM9414 (**A1**, **A2**) and Δcre1 (**B1**, **B2**). Precultures were inoculated with 10^5^ spores/mL (**A1**, **B1**) or with a piece of overgrown agar (**A2**, **B2**). All cultivations were incubated at 190 rpm

**Fig. 2:**
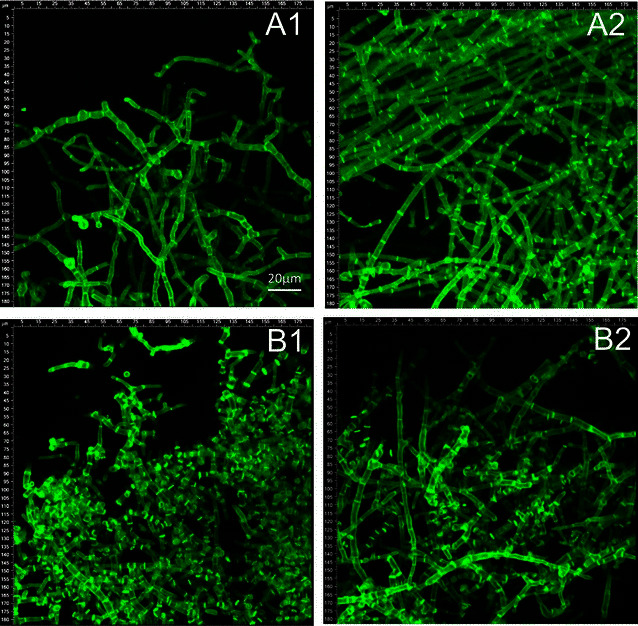
CLSM images of lactose cultivations by *T. reesei* strains QM9414 (**A1**, **A2**) and Δcre1 (**B1**, **B2**). Precultures were inoculated with 10^5^ spores/mL (**A1**, **B1**) or with a piece of overgrown agar (**A2**, **B2**). All cultivations were incubated at 190 rpm

The CLSM images shown in Figs. 1 and 2 reveal distinct micromorphological differences between strains QM9414 and Δcre1. To enable comparison of the two strains, the average cell length, cell width, and number of branches per septa were calculated for wheat straw and lactose cultivations, respectively. The results are also summarized in Table 1. Independent of the carbon source, Δcre1 formed shorter and wider cells than QM9414. Δcre1 further developed a more ramified mycelia with up to 2.25-fold more branches per cell (Table 1). Note that the removal of *creA*, the *cre1* orthologue gene in *A. nidulans*, also resulted in an increased degree of branching in submerged cultures of fungus [33].

#### Wheat straw and lactose cultivations

In cultivations on lactose, both strains produced 50 % less proteins on average than on wheat straw (Table 1; Additional file 5), and only basal amounts of filter paper activity were detected. This was in accordance with earlier proteomic studies, where it was shown that although lactose induced the expression of the complete set of hemicellulases and cellulases [17], the level of expression was much lower as compared to wheat straw [18].

In cultivations on wheat straw, strains QM9414 and Δcre1 developed 10 % and 21 % shorter cells on average as compared to lactose cultivations. In addition, the cells of Δcre1 were 10 % wider (Table 1). QM9414 also tended to form wider cells on wheat straw (Table 1). However, the changes were less pronounced.

#### Inoculation method

**Fig. 3:**
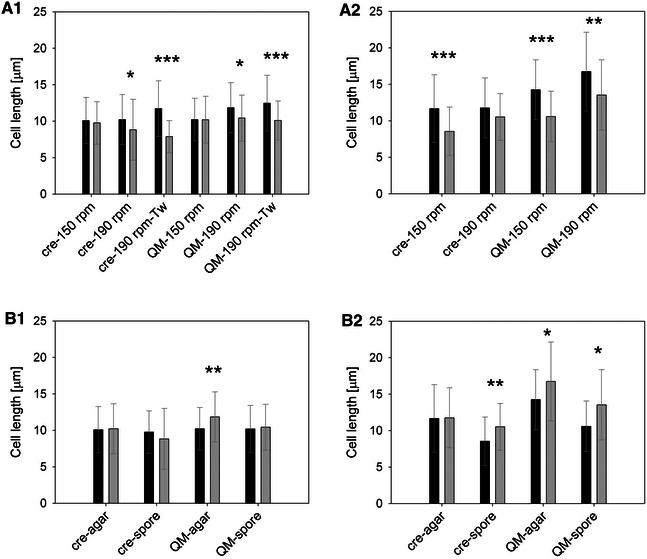
Influence of the inoculation method (**A1**, **A2**) and the agitation velocity (**B1**, **B2**) on cell length. Cell length was analyzed in wheat straw (**A1**, **B1**) and lactose (**A2**, **B2**) cultivations. **A1**, **A2** Precultures were inoculated with a piece of overgrown agar (*black bars*) or 10^5^ spores/mL (*grey bars*). **B1**, **B2** Agitation velocity was 150 rpm (*black bars*) or 190 rpm (*grey bars*) in an orbital incubator shaker. Cultivations were accomplished with *T. reesei* strain Δcre1 (“cre”) or QM9414 (“QM”). Incubation was at 150 or 190 rpm, as indicated. The addition of Tween80 is marked with “Tw”. Data were taken from Table 1, where *bars* indicate the mean length and *error bars* show the spread. Data sets with 1, 2, or 3 stars mark significance in a 95, 99, and 99.9 % confidence level, respectively

Cultivations were either started by adding a piece of overgrown agar or by adding spores to a final concentration of 10^5^ spores/mL. With the piece of overgrown agar, pre-existing hyphal biomass was added to the cultivations. Because the fungus does not have to develop from the spores, this method results in faster onset of biomass formation. Consequently, 1.7-fold more biomass was obtained in agar- as compared to spore-inoculated cultivations (Additional file 5). In the literature, however, inoculation with spores has been the method of choice [8, 34–36]. To analyze the impact on the micromorphology, the cell length was compared in dependence of the inoculation method and the results are shown in Fig. 3 (panels A1, A2). Independent of substrate, agitation velocity and strain used, agar-inoculated cultivations resulted in longer septa as compared to spore-inoculated cultures (Fig. 3, panels A1, A2). It is noteworthy that the micromorphological analysis was conducted from samples of the main cultures. Thus, the shift in micromorphology caused by the inoculation method was sustained over the precultures and was still detectable in the main cultivations.

The inoculation method further affected the degree of branching (Figs. 1, 2), as well as the cellulase production (Table 1). Therefore, in spore-inoculated cultivations, QM9414 and Δcre1 formed averagely 5 and 8 % more branches per cell as compared to agar-inoculated cultivations. When wheat straw cultivations were inoculated with spores, QM9414 and Δcre1 produced 1.4 and 2 FPU/mL on average (Table 1). This is 1.7- and 1.4-fold higher as compared to agar-inoculated cultivations by QM9414 (0.8 FPU/mL) and Δcre1 (1.4 FPU/mL), respectively. Protein secretion has been suggested to primarily happen at the tip of the apical compartment [37, 38]. The higher degree of branching and the corresponding increase in freshly formed tips, therefore, likely caused the observed increase in enzyme production.

#### Agitation velocity

Because of its impact on oxygen input, morphology, and fragmentation, the type and intensity of agitation are a key parameter for cultivations by filamentous fungi [11, 12, 39–43]. To analyze the impact of the agitation velocity on the micromorphology of strains QM9414 and Δcre1, cultivations were incubated at 190 and 150 rpm. The resulting cell lengths are compared in Fig. 3 (panels B1, B2). In cultivations on wheat straw, no clear effect of the agitation velocity on the cell length was observed. On lactose, however, incubation at 190 rpm seemingly resulted in longer cells as compared to 150 rpm (Fig. 3).

It has been shown that the length of the apical compartment increases with increasing biomass growth rates [44]. The longer cells observed at 190 rpm might, therefore, be a result of the increased oxygen transfer rate and the resulting higher biomass growth. An increase in the intercalary cell length with higher stirring rates has been also described for *A. nidulans* strains [45].

Wheat straw cultivations showed a much higher viscosity because of the solids load and the dispersed macromorphology (Additional file 3). The difference between 150 and 190 rpm was, therefore, probably not enough to influence the oxygen transfer rate to the extent necessary to induce changes in the cell length (Fig. 3).

#### Tween80

The influence of the surface-active agent Tween80 on the micromorphology and enzyme production was analyzed in wheat straw cultivations. In agar-inoculated cultivations, both the strains tended to develop longer cells and a lower filter paper activity when Tween80 was added to the culture media (Table 1). In spore-inoculated cultivations, Δcre1 developed shorter cells and produced more FPU/mL when Tween80 was added. QM9414 developed longer cells in spore-inoculated cultivations with supplemented Tween80 and a higher cellulase activity (Table 1). It has been described that Tween80 increases the permeability of the cell membrane. This facilitates protein secretion, and thus positively influences the cellulase production by *T. reesei* [7, 8, 46]. In this study, however, no clear effect of Tween80 on the micromorphology or the enzyme production could be observed (Table 1).

### Micromorphological changes at single cell level affect the protein and cellulase production

**Fig. 4:**
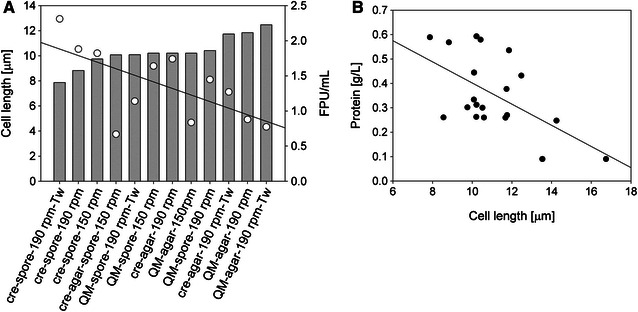
Correlation between cell length and total cellulase activity in wheat straw cultivations (**A**) and total protein concentration in wheat straw and lactose cultivations (**B**). **A** Depicted are cell length (*grey bars*) and total cellulase activity (*empty circles*). Cultivations were accomplished with *T. reesei* strain Δcre1 (“cre”) or QM9414 (“QM”). Inoculation was with a piece of overgrown agar (“agar”) or 10^5^ spores/mL (“spores”). Incubation was at 150 and 190 rpm, as indicated. The addition of Tween80 is marked with “Tw”. **B** Depicted are cell length and the corresponding protein concentration in the supernatant of all experimental setups. Data are taken from Table 1

As presented above, the dimensions of the cells were varying in dependence of strain, substrate, and process conditions used. In Fig. 4 (panel A), the cell length and the total cellulase activity (FPU/mL) of all presented wheat straw cultivations are compared. The results were sorted according to the measured cell lengths, and it appeared as both the strains tended to produce more cellulases in cultivations when shorter cells were formed. The cellulase activity (FPU/mL) was also plotted over the corresponding cell length, and the regressed change in filter paper activity with increasing cell length was −0.30 FPU/(mL µm) (*R* ^2^ 0.53). To include lactose cultivations, where low protein expression precluded measurement of the filter paper activity, the protein concentration of all experimental setups was plotted against the cell length. The results are shown in Fig. 4 (panel B). Regardless of strain, carbon source, agitation velocity, or inoculation method used, the protein concentrations were higher in cultivations that elicited shorter cells. The protein concentration correlates with the cell length with a regression coefficient of −0.04 g/(L µm) (*R* ^2^ 0.33). This was in accordance with the observed trend for cultivations on wheat straw (Fig. 4, panel A), and it can be concluded that there was a negative correlation between the cell length and the protein secretion.

**Fig. 5:**
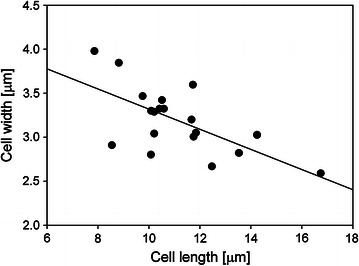
Correlation between cell length and cell width. Data are taken from Table 1 and include all experimental setups

Filamentous fungi grow highly polarized by extension of the apical compartment [6]. The extension proceeds until the cell is through an active mitotic cell cycle in which the multiple nuclei duplicate, and stops with the septation of the compartment [44, 47]. The size of the apical compartment is thereby variable and dependent on the maximal growth rate, where faster growth is linked to a longer apical cell [44]. It has been further observed that, with an increase in biomass growth, the hyphae diameter is also increasing [44, 45, 48]. A higher biomass growth rate, in turn, can result in higher protein productivities [48]. A positive correlation between cellulase productivity and hyphae diameter has been described for cultivations by *T. reesei* [7]. In this study, a decrease in cell lengths was accompanied by an increase in cell diameter (Fig. 5, regression coefficient −0.11 µm/µm, *R* ^2^ 0.42). The shorter, and consequently wider cells, observed in cultivations, where cellulase production was high (Fig. 4), might, therefore, indicate that the fungal strains grew at faster rates, which resulted in increased protein productivities [7, 48]. It has been further shown that the branching mainly occurs at the first subapical compartment [38]. The shorter cells and the corresponding faster septation might, therefore, have induced the formation of more branches [38]. A higher degree of branching that is accompanied by an increase in freshly formed hyphae tips has been described to increase protein secretion [37, 38].

## Conclusions

In this study, we present for the first time, the analysis and quantification of the micromorphology of *T. reesei* growing on a solid lignocellulosic substrate. The micromorphology was strongly dependent on strain, carbon source, and inoculation method used. Changes in cell length appeared to correlate with the protein (−0.04 g/(L µm); *R* ^2^ 0.33) and cellulase production (−0.30 FPU/(mL µm), *R* ^2^ 0.53). Consequently, in Tween supplemented wheat straw cultivations using ∆cre1, where the highest cellulase activity (2.31 FPU/mL) was achieved, the shortest (~7.9 µm) and widest (~4.0 µm) cells were measured. Micromorphology at the single cell level, appears to affect the enzyme productivity in *T. reesei*. The herein presented method, provides a useful tool to analyze and optimize cultivations of *T. reesei* on lignocellulosic feedstocks.

### Additional files

**Additional file 1.** The interface of the MATLAB program (A) and an example of the resulting 3-dimensional hyphae skeleton (B). A: A screenshot of the MATLAB program for processing the CLSM images with a short description of the control elements. B: A 3-dimensional representation of the hyphae skeleton after the image processing with the MATLAB program.

**Additional file 2.** Cell length distribution. Examples of the normal distribution of the cell lengths in wheat straw and lactose cultivations as indicated.

**Additional file 3.** Differences in macromorphology in cultivations on lactose (A1 and B1) and wheat straw (A2 and B2). Depicted are cultivations by *T. reesei* strains Δcre1 (A1 and A2) and QM9414 (B1 and B2), directly inoculated with 10^5^ spores/mL and incubated at 190 rpm.

**Additional file 4.** CLSM image of fungal growth on wheat straw. Cultivations on wheat straw by *T. reesei* strain QM9414. The culture was directly inoculated with a piece of overgrown agar and incubated at 190 rpm.

**Additional file 5.** Protein (A and B) and biomass (C) production in cultivations by *T. reesei* strains Δcre1 and QM9414. A and B: Depicted are the protein concentrations over time in wheat straw (A) and lactose (B) cultivations by *T. reesei* strains Δcre1 (circles) and QM9414 (triangles). Cultures were directly inoculated with 10^5^ spores/mL (open symbols) or a piece of overgrown agar (closed symbols). C: The final biomass (wet weight) concentration was measured in lactose cultivations after 200 h of incubation. Cultivations were directly inoculated with a piece of overgrown agar (black bars) or 10^5^ spores/mL (grey bars). Incubation for all experiments was at 190 rpm.

**Additional file 6.** Cellulase activity, β-glucosidase activity and protein concentration in wheat straw cultivations by *T. reesei* strains Δcre1 and QM9414. Depicted are the total cellulase activity (FPU/mL, black bars), the β-glucosidase activity (CBU/mL, light grey bars) and the protein concentration (dark grey bars) obtained from cultivations on wheat straw and lactose as indicated.
