# Supplementary figures and images for: The micromorphology of Trichoderma reesei analyzed in cultivations on lactose and solid lignocellulosic substrate, and its relationship with cellulase production
Source: Biotechnol Biofuels. 2016 Aug 9;9:169. doi: 10.1186/s13068-016-0584-0 (PMC4979124; doi:10.1186/s13068-016-0584-0)

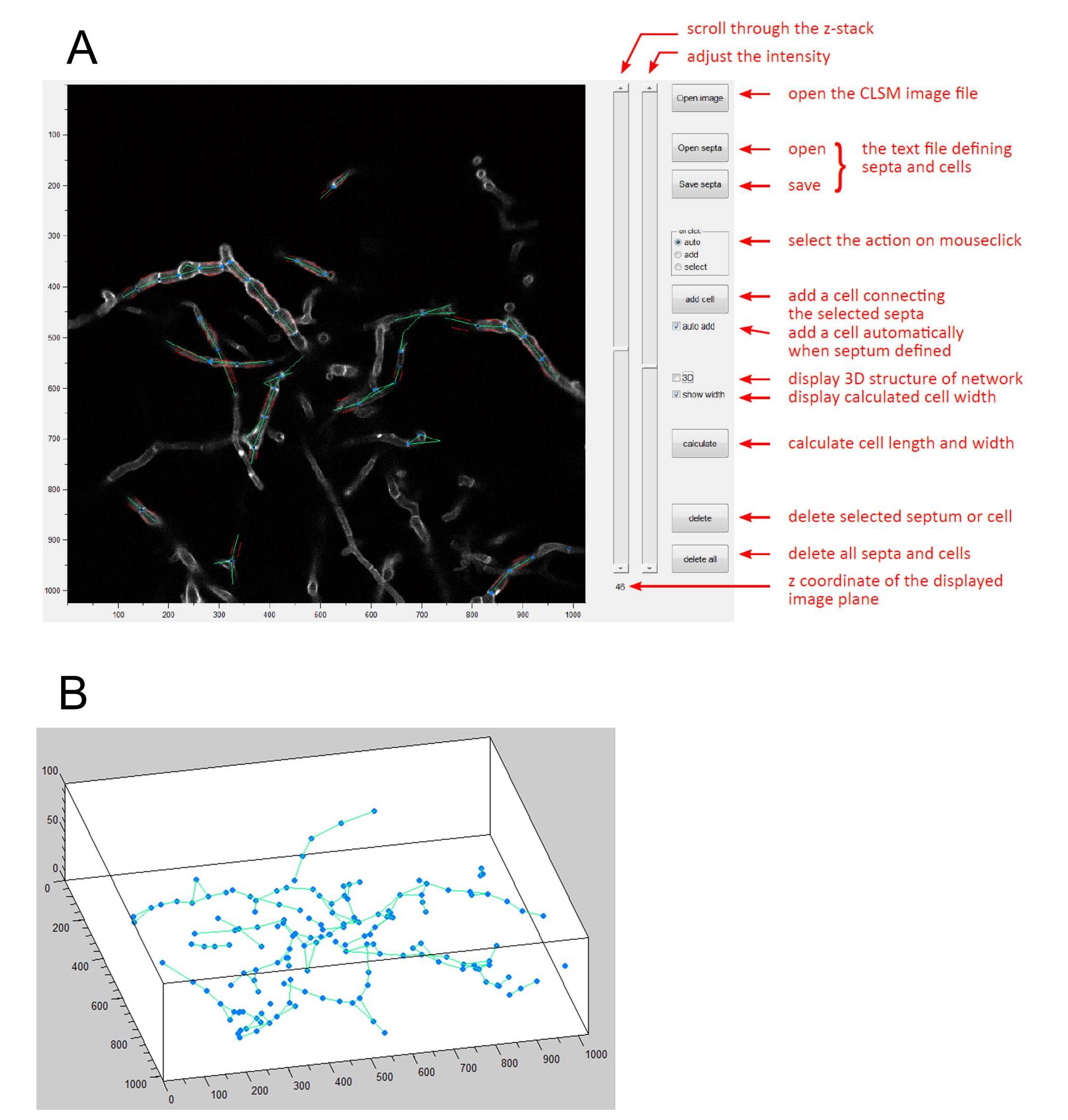

Supplement: Supplementary file 1 — Additional file 1. The interface of the MATLAB program (A) and an example of the resulting 3-dimensional hyphae skeleton (B). A: A screenshot of the MATLAB program for processing the CLSM images with a short description of the control elements. B: A 3-dimensional representation of the hyphae skeleton after the image processing with the MATLAB program. [file 13068_2016_584_MOESM1_ESM.jpg]

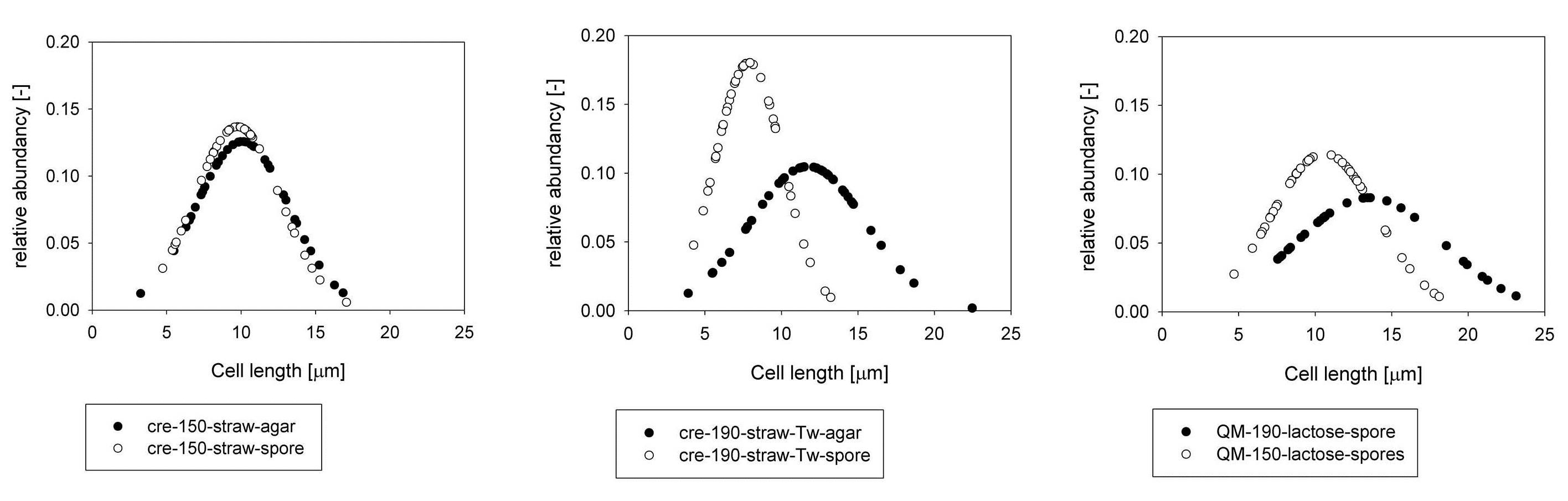

Supplement: Supplementary file 2 — Additional file 2. Cell length distribution. Examples of the normal distribution of the cell lengths in wheat straw and lactose cultivations as indicated. [file 13068_2016_584_MOESM2_ESM.jpg]

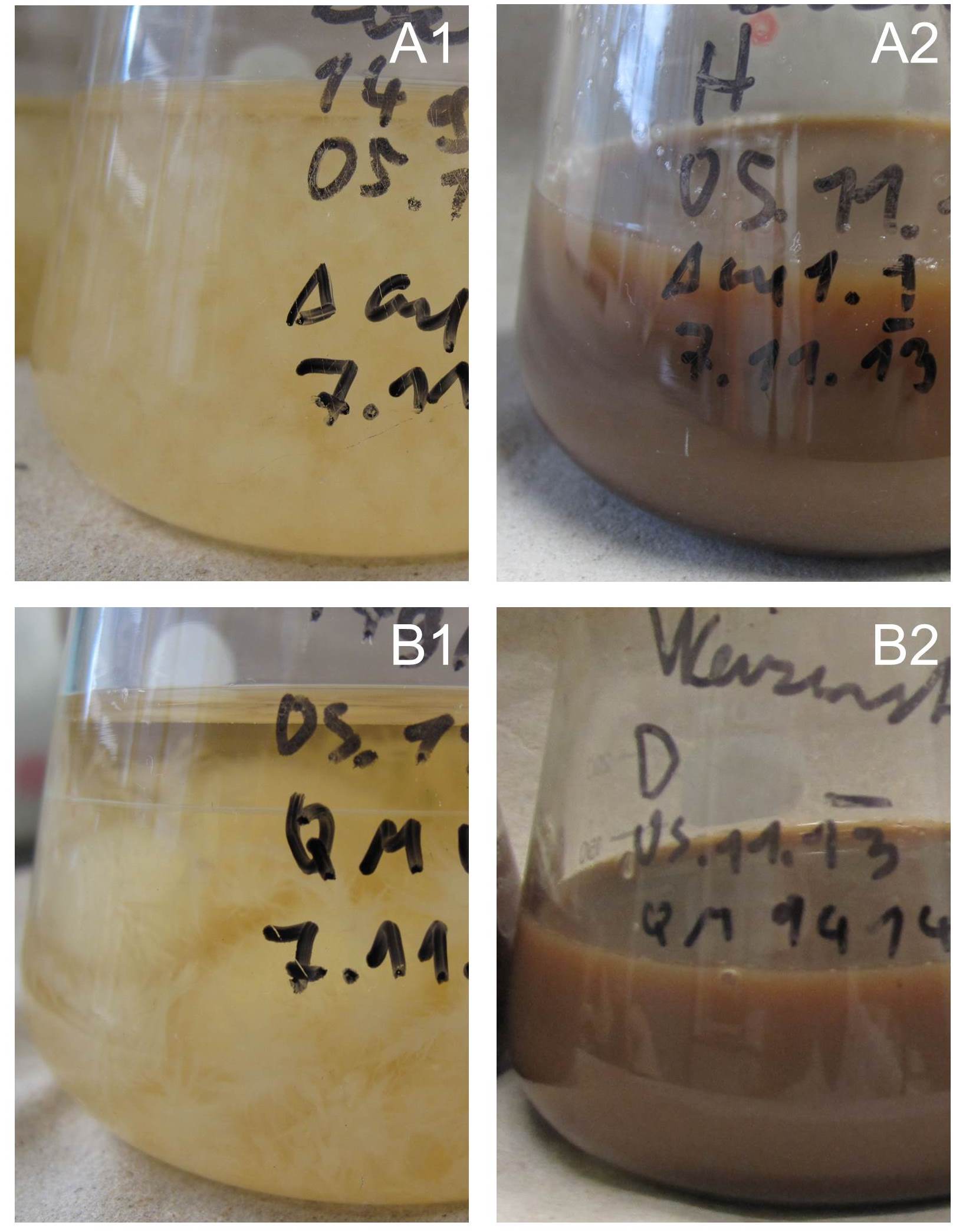

Supplement: Supplementary file 3 — Additional file 3. Differences in macromorphology in cultivations on lactose (A1 and B1) and wheat straw (A2 and B2). Depicted are cultivations by T. reesei strains Δcre1 (A1 and A2) and QM9414 (B1 and B2), directly inoculated with 105 spores/mL and incubated at 190 rpm. [file 13068_2016_584_MOESM3_ESM.jpg]

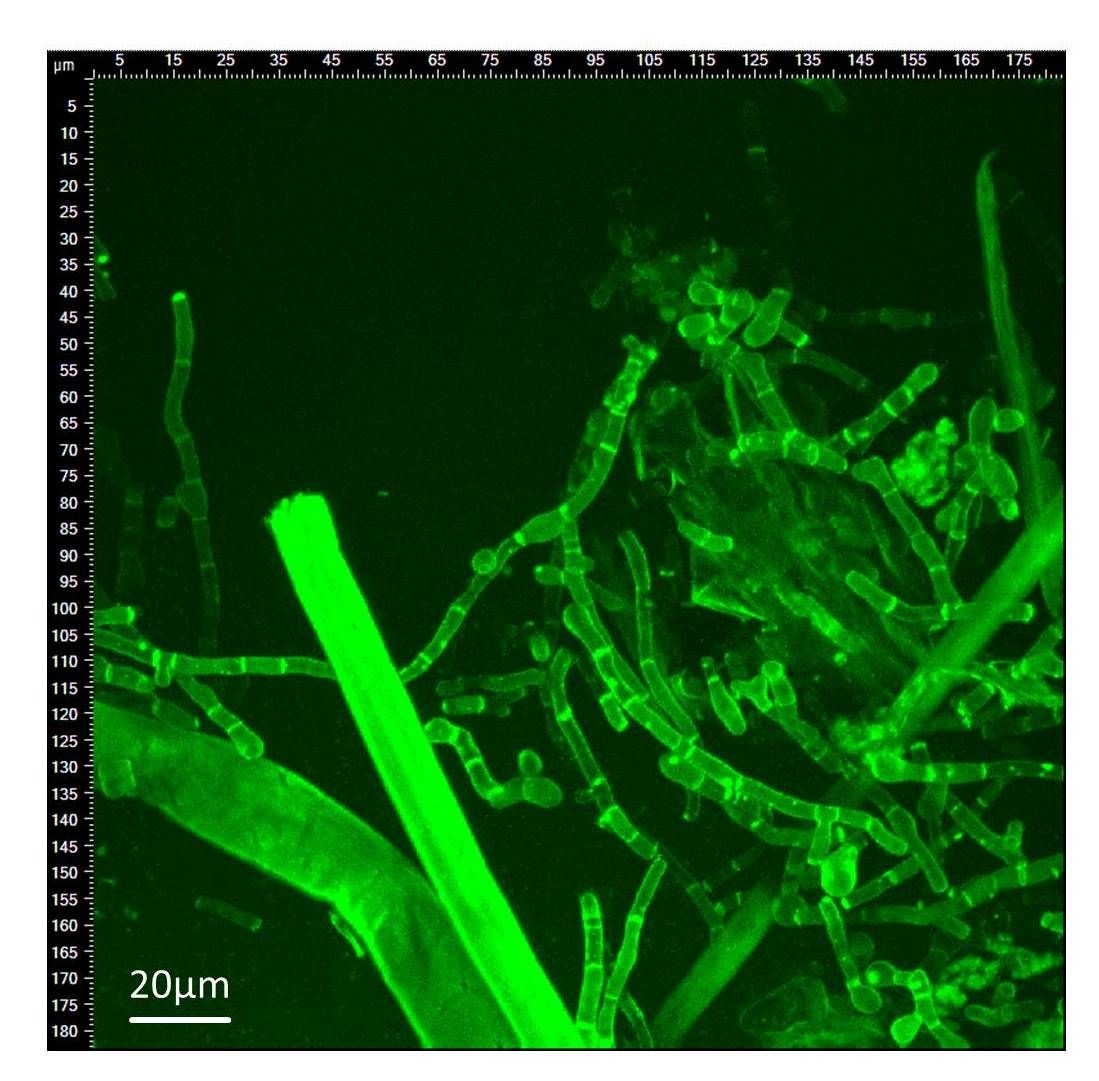

Supplement: Supplementary file 4 — Additional file 4. CLSM image of fungal growth on wheat straw. Cultivations on wheat straw by T. reesei strain QM9414. The culture was directly inoculated with a piece of overgrown agar and incubated at 190 rpm. [file 13068_2016_584_MOESM4_ESM.jpg]

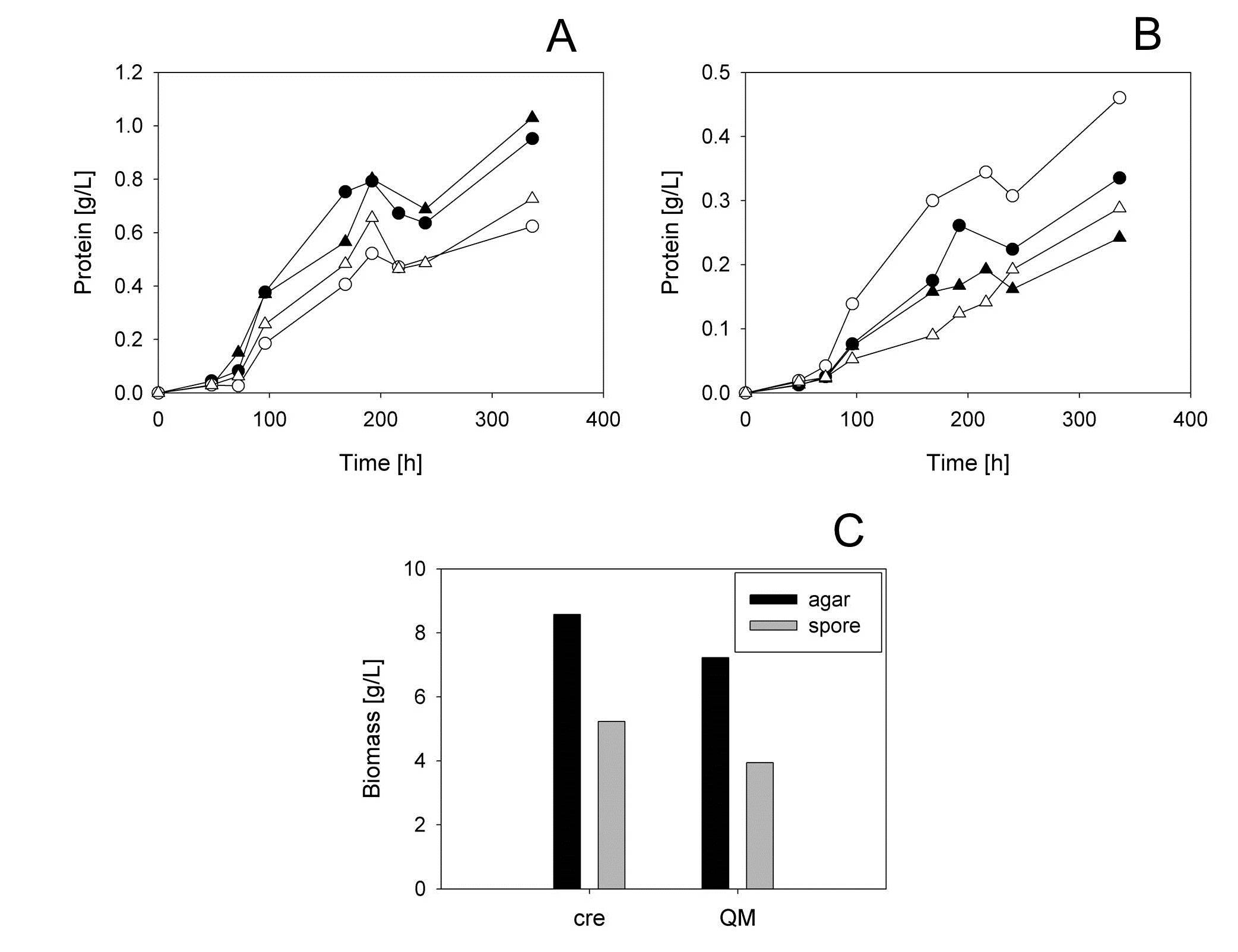

Supplement: Supplementary file 5 — Additional file 5. Protein (A and B) and biomass (C) production in cultivations by T. reesei strains Δcre1 and QM9414. A and B: Depicted are the protein concentrations over time in wheat straw (A) and lactose (B) cultivations by T. reesei strains Δcre1 (circles) and QM9414 (triangles). Cultures were directly inoculated with 105 spores/mL (open symbols) or a piece of overgrown agar (closed symbols). C: The final biomass (wet weight) concentration was measured in lactose cultivations after 200 h of incubation. Cultivations were directly inoculated with a piece of overgrown agar (black bars) or 105 spores/mL (grey bars). Incubation for all experiments was at 190 rpm. [file 13068_2016_584_MOESM5_ESM.jpg]

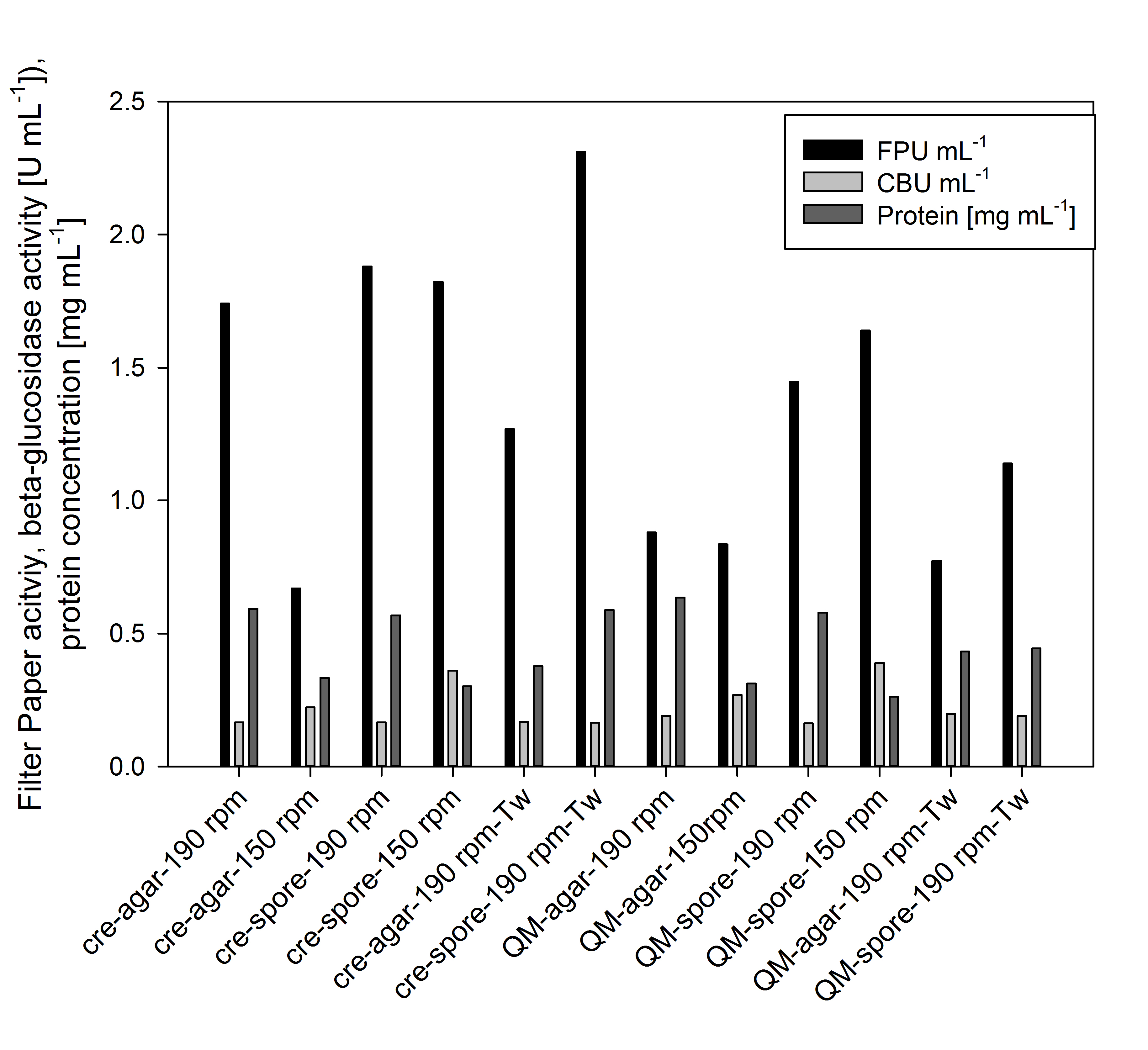

Supplement: Supplementary file 6 — Additional file 6. Cellulase activity, β-glucosidase activity and protein concentration in wheat straw cultivations by T. reesei strains Δcre1 and QM9414. Depicted are the total cellulase activity (FPU/mL, black bars), the β-glucosidase activity (CBU/mL, light grey bars) and the protein concentration (dark grey bars) obtained from cultivations on wheat straw and lactose as indicated. [file 13068_2016_584_MOESM6_ESM.jpg]
